# Cementless total hip arthroplasty for three different degrees of hip involved secondary to ankylosing spondylitis: an analysis of 195 hips

**DOI:** 10.1186/s13018-021-02742-6

**Published:** 2021-10-16

**Authors:** Ping Mou, Hua Li, An-Jing Chen, Zheng Ji, Xin-Yi Dai, Zong-Ke Zhou

**Affiliations:** 1 Department of Orthopedics, Orthopedic Research Institute, West China Hospital, Sichuan University, #37 Guoxue Road, Chengdu, 610041 People’s Republic of China; 2 Department of Rehabilitation Medicine, Jiang You Second People’s Hospital, #10 Tuanshan Road, Jiang you, 621702 People’s Republic of China; 3 West China School of Nursing, Sichuan University, Chengdu, 610041 People’s Republic of China

**Keywords:** Total hip arthroplasty, Ankylosing spondylitis, Ankylosed hip, Non-ankylosed hip, Range of motion

## Abstract

**Background:**

Hip involved secondary to ankylosis spondylitis (AS) had a huge influence on hip function. Cementless total hip arthroplasty (THA) can improve hip function. However, no previous study compared the outcomes of THA for AS patients with three different degrees of hip involvement.

**Methods:**

The 195 hips were retrospectively analyzed and divided into non-ankylosed group (group A, 94 hips), fibrous ankylosed group (group B, 49 hips), and bony ankylosed group (group C, 52 hips). postoperative range of motion (ROM), harris hip scores (HHS), the short-form 12 health survey (SF-12), length of stay (LOS), cost, radiological assessments, and complications were compared.

**Results:**

The follow-up time was (79.4 ± 29.5) months for group A, (80.6 ± 28.9) months for group B, and (79.1 ± 28.9) months for group C (*P* = 0.966). Group A had the best postoperative hip ROM (*P* < 0.001), while group A and B can realize better HHS than group C (*P* < 0.001). The three groups had similar SF-12 postoperatively. For group A, LOS and cost for unilateral procedure were the least than that for group B and C (*P* = 0.003 and *P* = 0.001). Similar radiological assessments were achieved for three groups. 1 hip in group A encountered delay union of wound. 1 hip in group C encountered delay union of wound and dislocation and another patient encountered femoral fracture intraoperatively. 12 hips (12.8%) in group A, 6 hips (12.2%) in group B, and 6 hips (11.5%) in group C encountered asymptomatic heterotopic ossification (*P* = 0.977).

**Conclusion:**

For AS patients with hip involvement, THA can improve hip ROM and function. THA for the non-ankylosed hip can realize the better hip function and postoperative ROM than ankylosed hip.

## Introduction

Ankylosing spondylitis (AS) diagnosed using the modified New York criteria [1] is an inflammatory disease affecting the axial spine and peripheral joints, characterized by low back pain, limited range of motion (ROM) of the lumbar spine and hip joint involved, and resulting in functional impairment and decreased quality of life [2]. The diagnosis of AS based on the modified New York criteria requires sacroiliitis on imaging, low back pain and stiffness for > 3 months, limited lumbar spine ROM, and/or limited chest expansion. And the prevalence of AS in China is 0.22% and more common in males (0.36%) than in females (0.09%) but no significant regional difference [3]. Hips involved occupied 30–50% of patients diagnosed with AS [4]. The involved hips present with progressive pain, bone deformity, and gradual loss of hip ROM along with disease advance. Flexion contracture of hip and spinal kyphosis were the two most common characteristics for AS patients [5]. The mobility of the hip is conducive to compensatory for kyphosis, correction of limp, and functional improvement [6]. So, ROM restoration for AS patients is crucial.

The literature has reported that AS is diagnosed correctly for 7–10 years after patients experience various symptoms. Effective treatment may be delayed for the patients until correct diagnosis [7, 8] and the hips may progress to various degrees of involvement and present different degrees of ROM loss. Additionally, AS was pathologically characterized by inflammation of tendon insertion [2]. And different degrees of lesions caused by AS showed various changes of soft tissue, which may result in different influences on hip function even if total hip arthroplasty (THA) was performed. Currently, according to the different severities of hip lesions, three different lesions can emerge including non-ankylosed hip [6, 9, 10], fibrous ankylosed hip [11–13], and bony ankylosed hip [10–12, 14, 15], which were defined as no total loss of hip ROM, no continuous bony trabecula passing through the hip joint on X-ray with 0°hip ROM, and continuous bony trabecula bridging the hip joint on X-ray with 0°hip ROM. Moreover, different severities of lesions indicated different technological demands and operational invasions. The scholars [13] considered that degree of bone deformity and the level of soft tissue contracture were two decisive contributors to hip ROM. They [13] also stressed that rehabilitation training according to different deformities was related to postoperative ROM. Also, other scholars [6] concluded poor postoperative ROM in patients with preoperative bony ankylosis than that with stiff cases. Currently, literature has reported the clinical and radiographic outcomes of THA for AS patients. But they have different limitations such as small sample size [10–12, 14], short-term follow-up [5, 14], various implanted prostheses or fixation techniques [9, 15], and confounded results of bony and fibrous ankylosed hips [11, 12, 16]. No one study compared the outcomes of THA for three different degrees of hip involvement secondary to AS.

So, we retrospectively analyzed the AS patients in our joint center with different degrees of hip involvement and reported the effectiveness of THA for these patients. It was hypothesized that for AS patients with hip involvement, THA can improve hip function and realize good radiographic assessments for AS patients. And the differences may be found in three groups on clinical and radiographic evaluation.

## Materials and methods

### Ethics statement

Data were collected by retrospective review of our database from January 2010 to December 2017. Study approval was obtained from the Clinical Trials and Biomedical Ethics Committee of West China Hospital (ID: 2012-268). All experiments were performed following relevant guidelines and regulations. This study was conducted according to the Declaration of Helsinki. The data included patient demographics, clinical measurements, radiological assessments, and complications.

### Patients

The inclusion criteria: patients diagnosed with AS and performed cementless THA; hip involved secondary to AS; no total loss of hip ROM regarded as non-ankylosed group (group A); no continuous bony trabecula passing through the hip joint on X-ray with 0°hip ROM regarded as fibrous ankylosed group (group B); continuous bony trabecula passing through the hip joint on X-ray with 0°hip ROM regarded as bony ankylosed group (group C). The exclusion criteria: patients performed THA for other reasons; hip ankylosis caused by other reasons.

### Surgical procedures

After general anesthesia, all THA was performed using a posterolateral approach in the lateral decubitus position. The femoral neck was identified according to the lesser trochanter after total capsulectomy and cutting off the external rotators. For the non-ankylosed hip, the joint space can be distinguished clearly. Hip dislocation, osteotomy of the femoral head, and acetabular reaming can be done like general THA regularly after removal of osteophytes and scar. But for the fibrous and bony ankylosed hip, femoral neck osteotomy was conducted without hip dislocation due to total loss of hip ROM. Take care to avoid damage to the greater trochanter and posterior acetabulum. The acetabulum was deepened and widened in the medial direction using hemispherical reamers with gradual increases in diameters. The counterrotation technique was used to avoid over-reaming of the osteoporotic acetabulum. And the original joint plane was located by the foveal soft tissue and incomplete gray ossifying cartilage. The optimal cup size and cup inclination of the acetabulum prosthesis were identified by the preoperative project and intraoperative fluoroscopy. And the anteversion of the cup was confirmed with the indication of transverse acetabular ligament and the long axis of the body. The press-fit technique was conducted to insert the cementless acetabular implants. If the initial stability was not satisfactory, additional screws would be used to fix the cup before inserting the liner.

Sequentially larger reamers were used to enlarge the canal until the diaphyseal cortex was reached. Then femoral trial prosthesis was inserted to correct the leg length discrepancy (LLD), check the stability in all directions, and optimize the femoral offset. The cementless femoral prosthesis and femoral head were inserted. The hip stability and ROM were checked again to ensure postoperative mobility. At last, the external rotator muscles were restored in situ and the drainage was selected according to the time of operation and blood loss before incision suturing. If the hip can’t be passively abducted more than 15°, adductor tenotomy would be performed. All prostheses were used the same brand (DePuy Orthopadics, Marsaw IN) and friction couples were selected based on the patients’ financial status and activity levels.

### Perioperative regimen

Isometric exercises and positive motion were encouraged to conduct in bed immediately after recovering from anesthesia. Prophylactic intravenous antibiotics and low-molecular-weight heparin (LMWH) were systematically conducted. Half-dose LMWH (enoxaparin, 0.2 ml, 2000 IU) was routinely scheduled subcutaneously 6 h postoperatively, and a full dose (enoxaparin, 0.4 ml, 4000 IU) was given at 24-h intervals until discharge. After discharge, 10 mg of rivaroxaban was used orally for 10 days. Non-steroidal anti-inflammatory drugs were used to relieve pain and reduce the chance of heterotopic ossification (HO) for 2 weeks. For unbearable postoperative pain, additional painkillers by intravenous or intramuscular injection were added. The drainage tube was removed within 24 h. Routine clinical follow-up visits were conducted at 2 weeks, 4 weeks, 12 weeks, and 6 months after surgery and annually.

### Clinical measurements

Clinical details were recorded including hip ROM, Harris Hip Scores (HHS) preoperatively and postoperatively, Additionally, satisfaction levels and the short-form 12 health survey (SF-12) score postoperatively were also evaluated. The special ruler was used to measure the hip flexion, extension, and abduction positively in the supine position. HHS (the total score was 100) was used to evaluate the function of the hip [17] and rated as excellent (90–100), good (80–89), fair (70–79), and poor (< 70). Patients’ satisfaction was evaluated using a self-administered four-category scale (very satisfied, satisfied, somewhat unsatisfied, and unsatisfied). SF-12 score including physical component summary (PCS) and mental component summary (MCS) can act as a measurement reflecting the life quality recently [18]. Additionally, from our database, we collected the data including patient demographics at the time of surgery, component information, length of stay (LOS), and total hospital expense (THE). LOS was calculated how many nights the patients stayed in the hospital. THE was calculated how much Chinese yuan (¥) the patients spent on THA in hospital and presented in Chinese yuan (¥); currently, 1¥ = 0.154 United States dollars (USD, $).

### Radiological assessments

**Fig. 1:**
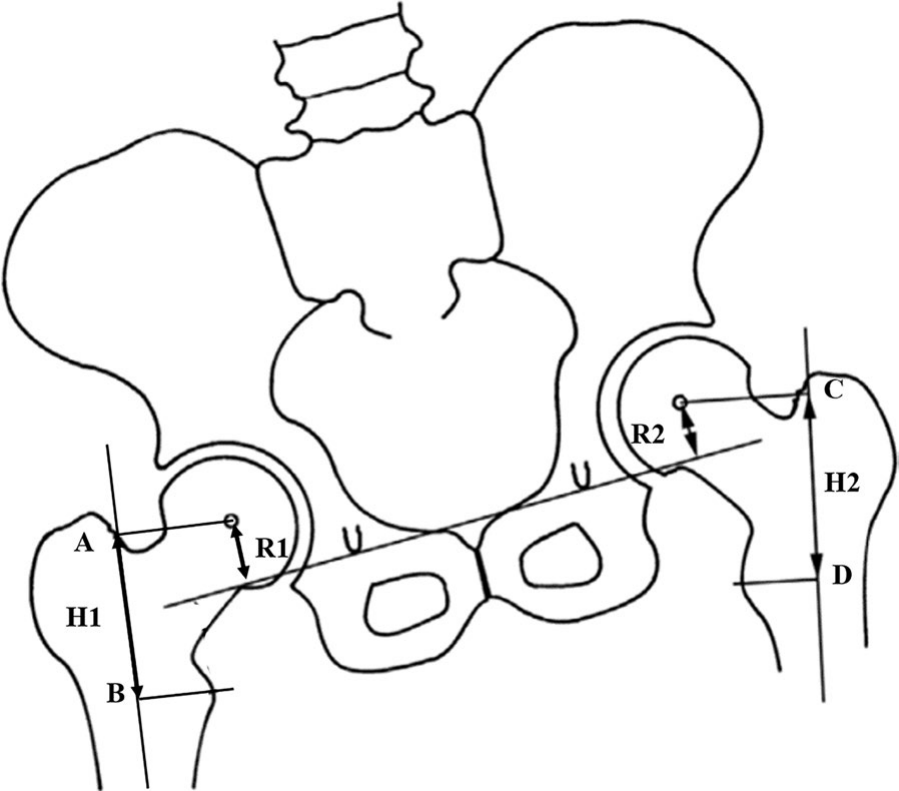
Diagram showing a standardized-trochanteric method to assess leg-length discrepancy. R1 and R2 are the vertical distance from the bilateral center of rotation to the inter-teardrop line. Line AB and line CD are the anatomical axes of the femurs. Point A and point C are the perpendicular intersections from the center of rotation to the femoral anatomical axis. Point B and point D are the perpendicular intersections from the tip of the lesser trochanter to the femoral anatomical axis. H1 and H2 are equal to AB and CD, respectively. Leg-length discrepancy = (H1 − R1) − (H2 − R2)

Standard anteroposterior (AP) radiograph was obtained preoperatively and postoperatively. The assessments included the inclination of the cup (IC), the femoral offset (FO), the difference of bilateral FO, and LLD. The angle crossed by the horizontal line connecting both teardrops and line through the longest diameter of the elliptical opening of the acetabular cup rim was recorded and regarded as IC [11]. If the teardrops were unrecognizable or the pelvis was asymmetric, we firstly bisected the sacrum with a vertical line A and secondly drawn a perpendicular line B to line A [11]. So, line B can be regarded as a horizontal line. FO was defined as the vertical distance from the center of the femoral head to the ipsilateral anatomical femoral axis [19]. LLD was assessed by the standardized-trochanteric method to avoid the influence of pelvic obliquity and femoral inclination on the radiographs [20]. The standardized-trochanteric method required the vertical distance from the inter-teardrop line to the center of rotation and the femoral vertical distance (center of rotation to the lesser trochanter) reference to the femoral anatomical axis. So, the unilateral distance was defined as the difference between the two vertical distances. And LLD was equal to the difference between the two unilateral distances. Figure 1 has shown the detailed process of measurement.

### Complications

The complications were recorded including early-onset and late-onset complications. The possible early-onset complications collected from the database consisted of dislocation, wound complications, infection, intraoperative fracture, deep venous thrombosis, pulmonary embolism, and nerve palsy. Additionally, the possible late-onset complications consisted of dislocation, HO, osteolysis, and aseptic loosening. According to Brooker classification [21], we analyzed and classified the degrees of HO. Osteolysis was defined as cystic or scalloped lesions with a diameter of more than 2 mm on radiograph [22, 23]. According to the criteria of DeLee [24], the acetabular component was defined as loose if a complete radiolucent line was thicker than 2 mm at the bone-implant interface or migration of the component. Besides, the femoral implant stability was evaluated according to Engh et al. [25], the stem was considered loose if subsidence more than 2 mm or angular shift of the stem more than 2°.

For the clinical measurement, radiological assessment, and complication evaluation, two investigators (HL and AJC) who did not know the group allocation and were not involved in the surgery individually performed the measurements. If no consensus was got, the investigator (PM) who also did not know the group allocation confirmed the final results.

### Statistical analysis

Continuous data are presented as the mean and standard deviation (SD) or as the median with the interquartile range. Categorical data are shown as the number and percentage. A 1-way analysis of variance (ANOVA) with post-hoc Tukey test was used for normally distributed continuous variables, and the Kruskal–Wallis analysis with post-hoc Nemenyi test was used for skewed continuous variables. Chi-square or Fisher tests were applied for categorical variables. Significance was set at the level of *P* < 0.05.

## Results

Ultimately, we included 69 patients (94 hips) with the mean age of (42.2 ± 13.8) years old for group A, 40 patients (49 hips) with the mean age of (40.6 ± 13.3) years old for group B, and 28 patients (52 hips) with the mean age of (38.8 ± 8.5) years old for group C (*P* = 0.311). Among the patients, 11 patients from group A, 2 patients from group B, and 12 patients from group C were performed bilateral THA synchronously. And 14 patients from group A, 7 patients from group B, and 12 patients from group C were performed bilateral THA sequentially. And the others were performed unilateral THA. The positive rate of human leukocyte antigen B27 (HLA B27) was 84.1% for group A, 87.5% for group B, and 85.7% for group C (*P* = 0.885). Friction couples including ceramic-on-ceramic bearing (CoC) and ceramic-on-polyethylene bearing (CoP) were identical for all hips. The CoC bearing was used 64 hips (68.1%) for group A, 41 hips (83.7%) for group B, and 37 hips (71.2%) for group C, while CoP bearing was used 30 hips (31.9%) for group A, 8 hips (16.3%) for group B, and 15 hips (28.8%) for group C (*P* = 0.132). Table 1 showed the demographics of three study groups.

**Table 1:** Perioperative demographics and component information of all included patients

Variable	Group A	Group B	Group C	*P* value
*Demographic characteristics*				
M/F	58/11	38/2	24/4	0.342
L/R	54/40	22/27	26/26	< 0.001*
Age (years)	42.2 ± 13.8	40.6 ± 13.3	38.8 ± 8.5	0.311
Height (cm)	163.5 ± 7.9	165.6 ± 7.2^a^	161.0 ± 6.7	0.016*
Weight (kg)	60.4 ± 8.7	61.6 ± 8.4	58.1 ± 8.0	0.155
BMI (kg/m^2^)	22.6 ± 2.8	22.4 ± 2.4	22.4 ± 2.8	0.916
HLA B27 positive	58 (84.1%)	35 (87.5%)	24 (85.7%)	0.885
Average follow-up time (months)	79.4 ± 29.5	80.6 ± 28.9	79.1 ± 28.9	0.966
Bearing surfaces (no. of hips)				0.132
CoC bearing	64 (68.1%)	41 (83.7%)	37 (71.2%)	
CoP bearing	30 (31.9%)	8 (16.3%)	15 (28.8%)	

Values are expressed as mean ± SD or number or number of cases (percentage)

*P* values with statistical significance are marked with *

*M* male, *F* female, *L* left, *R* right, *BMI* body mass index, *HLA B27* human leukocyte antigen B27, *CoC* ceramic-on-ceramic bearing, *CoP* ceramic-on-polyethylene bearing

^a^Significantly different from group C

### Clinical outcomes

The mean postoperative follow-up was (79.4 ± 29.5) months for group A, (80.6 ± 28.9) months for group B, and (79.1 ± 28.9) months for group C, respectively (*P* = 0.966). The average HHS increased from (31.5 ± 4.3) to (89.1 ± 3.2) for group A, (30.9 ± 3.8) to (88.2 ± 3.2) for group B, and (30.9 ± 5.0) to (83.7 ± 2.3) for group C. Postoperative HHS of group A and group B was higher than that of group C (*P* < 0.001), while no difference was found between group A and group B (*P* = 0.078). The HHS of all patients from three groups was rated as poor preoperatively, while more than 90% of hips were rated as excellent or good at the latest follow-up. For group A, the flection-extension ROM was (72.2 ± 27.5)°. Moreover, significant difference was also found in preoperative flexion contracture and flexion among three groups (*P* < 0.001). At the latest follow-up, the average flection-extension ROM was (106.2 ± 9.9)° for group A, (102.3 ± 9.1)° for group B, and (84.1 ± 4.9)° for group C. Postoperative ROM of the three groups was different from that of each other (*P* < 0.001) (Table 2). For satisfaction levels, almost all patients were very satisfied with the outcomes and no difference was found between groups (*P* = 0.546). For the PCS and the MCS, no difference was found (*P* = 0.441 and *P* = 0.429) (Table 2).

**Table 2:** Clinical outcomes and functional evaluation of all included patients preoperatively and at the latest follow-up

Variable	Group A	Group B	Group C	*P* value
Pre-HHS	31.5 ± 4.3	30.9 ± 3.8	30.9 ± 5.0	0.641
Rating (no. of hips)				0.892
Excellent (90–100 points)	0 (0%)	0 (0%)	0 (0%)	
Good (80–89 points)	0 (0%)	0 (0%)	0 (0%)	
Fair (70–79 points)	0 (0%)	0 (0%)	0 (0%)	
Poor (< 70 points)	94 (100%)	49 (100%)	52 (100%)	
Post- HHS	89.1 ± 3.2 ^b^	88.2 ± 3.2 ^b^	83.7 ± 2.3	< 0.001*
Rating (no. of hips)				< 0.001*
Excellent (90–100 points)	45 (47.9%)	13 (26.5%)	0 (%)	
Good (80–89 points)	47 (50%)	35 (71.4%)	48(92.3%)	
Fair (70–79 points)	2 (2.1%)	1 (2.0%)	4 (7.7%)	
Poor (< 70 points)	0 (0%)	0 (0%)	0(0%)	
Pre- ROM (°)	72.2 ± 27.5 ^a, b^	0	0	< 0.001*
Pre- flexion contracture (°)	7.3 ± 11.6 ^a, b^	23.6 ± 20.3 ^b^	38.1 ± 19.1	< 0.001*
Pre- flexion (°)	79.5 ± 23.8 ^a, b^	23.6 ± 20.3 ^b^	38.1 ± 19.0	< 0.001*
Post- ROM (°)	106.2 ± 9.9 ^a, b^	102.3 ± 9.1 ^b^	84.1 ± 4.9	< 0.001*
Post- flexion contracture (°)	0.8 ± 2.2	0.9 ± 2.4	1.3 ± 3.1	0.457
Post- flexion (°)	106.9 ± 9.5 ^a, b^	103.3 ± 8.7 ^b^	85.5 ± 4.7	< 0.001*
Post- abduction (°)	33.7 ± 3.6	33.5 ± 4.4	32.6 ± 4.0	0.244
Satisfication level				0.546
Very satisfied	80 (96.4%)	45(95.7%)	39 (97.5%)	
Satisfied	3 (3.6%)	2(4.3%)	1 (2.5%)	
Somewhat unsatisfied	0 (0%)	0 (0%)	0 (0%)	
Unsatisfied	0 (%)	0 (%)	0 (%)	
SF-12				
PCS	22.1 ± 1.8	22.1 ± 1.8	21.6 ± 2.3	0.441
MCS	25.4 ± 2.1	24.9 ± 2.2	25.0 ± 1.9	0.429

Values are expressed as mean ± SD

*P* values with statistical significance are marked with *

*Pre-* preoperative, *Post-* postoperative, *HHS* Harris hip score, *ROM* range of motion, *PCS* physical component summary, *MCS* mental component summary

^a^Significantly different from group B

^b^Significantly different from group C

LOS for bilateral synchronous THA was (9.8 ± 1.6) days for group A, (14.0 ± 1.4) days for group B, and (16.8 ± 7.1) days for group C. The significant difference was found on LOS for bilateral synchronous THA between group A and group C (*P* = 0.003). No difference was found between group B and group C (*P* = 0.477) (Table 3). And LOS for unilateral THA was (9.3 ± 3.1) days for group A, (11.2 ± 3.9) days for group B, and (11.5 ± 3.7) days for group C. The significant difference was found on LOS for group A compared with group B and group C (*P* = 0.004 and *P* = 0.005). No difference was found between group B and group C (*P* = 0.729) (Table 3). Additionally, THE for bilateral synchronous THA was (108,481.5 ± 11,378.3) ¥ for group A, (126,640.5 ± 13,609.7) ¥ for group B, and (109,113.8 ± 6334.8) ¥ for group C. No difference was found among the three groups (*P* = 0.053) (Table 3). THE for unilateral THA was (54,520.4 ± 4377.6) ¥ for group A, (57,353.0 ± 3675.7) ¥ for group B, and (56,651.7 ± 3861.0) ¥ for group C. The significant difference was found on THE for group A compared to group B and C (*P* < 0.001 and *P* = 0.020) (Table 3).

**Table 3:** Length of stay and total hospital expense of all included patients

Variable	Group A	Group B	Group C	*P* value
*LOS*				
LOS for bilateral synchronous procedure (d)	9.8 ± 1.6^b^	14.0 ± 1.4	16.8 ± 7.1	0.012*
LOS for unilateral procedure (d)	9.3 ± 3.1^a,b^	11.2 ± 3.9	11.5 ± 3.7	0.003*
*THE*				
THE for bilateral synchronous procedure (¥)	108,481.5 ± 11,378.3	126,640.5 ± 13,609.7	109,113.8 ± 6334.8	0.053
THE for unilateral procedure (¥)	54,520.4 ± 4377.6^a,b^	57,353.0 ± 3675.7	56,651.7 ± 3861.0	0.001*

Values are expressed as mean ± SD

*P* values with statistical significance are marked with *

LOS, length of stay; THE, total hospital expense

^a^Significantly different from group B

^b^Significantly different from group C

### Radiographic evaluation

**Fig. 2:**
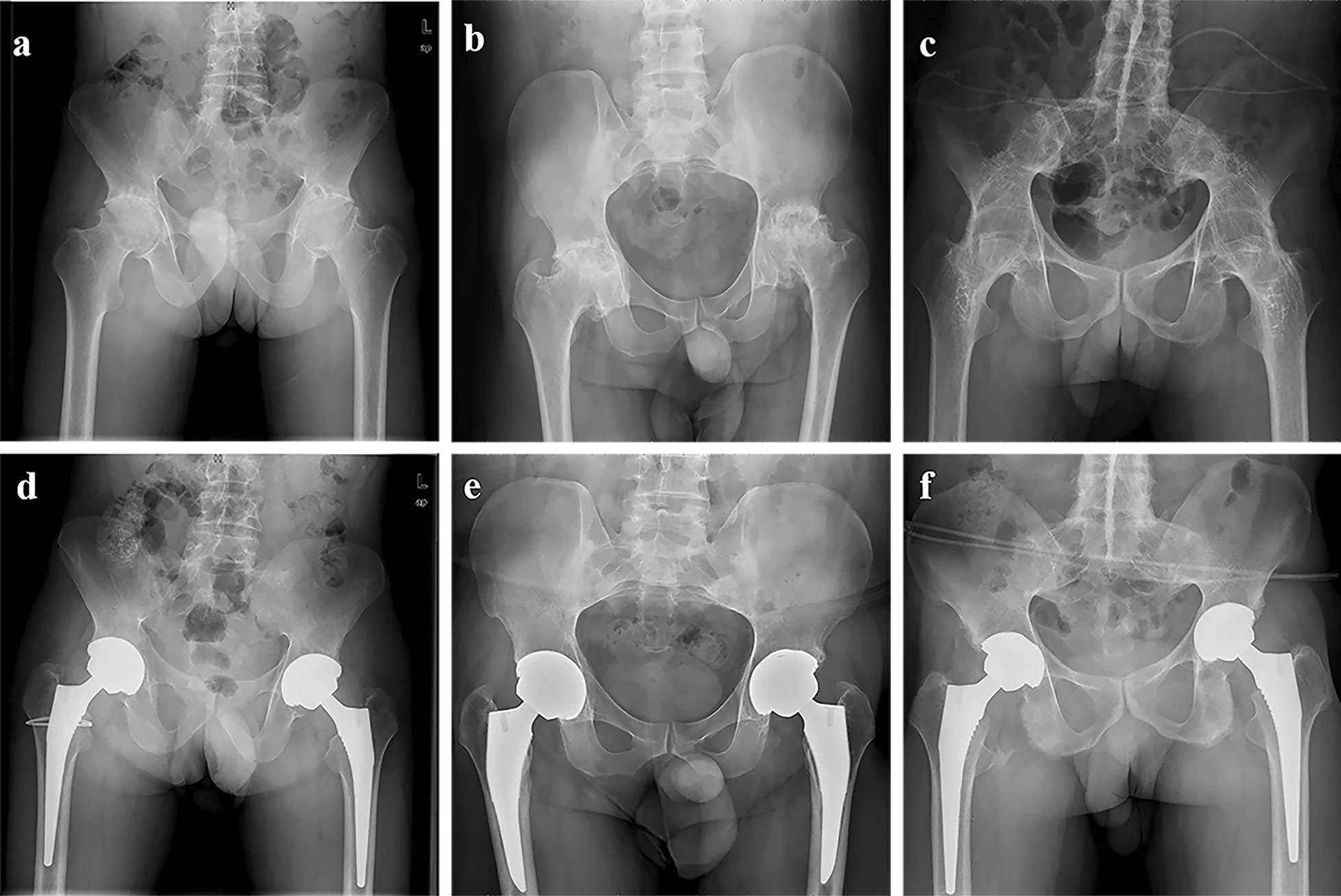
Case presentation of bilateral cementless bilateral total hip arthroplasty for three different degrees of hips involvement with ankylosing spondylitis. **a** The radiograph showed bilateral hips involved preoperatively and physical examination showed no total loss of range of motion. **b** The radiograph showed bilateral hips involved and no trabecula bridged the hip joint plane preoperatively and physical examination showed total loss of range of motion. **c** The radiograph showed bilateral hips involved and trabecula bridged the hip joint plane preoperatively and physical examination showed total loss of range of motion. **d** The pelvis radiograph at the latest follow-up (78 months for both hips) for Fig. 1a showed excellent prosthesis position, no loosening and migration of the component. **e** The pelvis radiograph at the latest follow-up (82 months for right hip and 81 months for left hip) for Fig. 1b showed excellent prosthesis position, no loosening and migration of the component. **f** The pelvis radiograph at the latest follow-up (80 months for both hips) for Fig. 1c showed excellent prosthesis position, no loosening and migration of the component

At the latest follow-up, the average IC was (39.5 ± 4.6)° for group A, (39.2 ± 4.3)° for group B, and (39.6 ± 4.6)° for group C (*P* = 0.882) (Table 4). The average FO was (4.38 ± 0.52) cm for group A, (4.23 ± 0.56) cm for group B, and (4.26 ± 0.52) cm for group C (*P* = 0.178) (Table 4). Moreover, the average difference of bilateral FO was (0.31 ± 0.21) cm for group A, (0.32 ± 0.18) cm for group B, and (0.34 ± 0.24) cm for group C (*P* = 0.794) (Table 4). And for LLD, the average LLD was (0.46 ± 0.31) cm for group A, (0.44 ± 0.34) cm for group B, and (0.44 ± 0.32) cm for group C (*P* = 0.940) (Table. 4). No difference was found on the IC, the FO, the difference of IC, and LLD. Figure 2 has shown the radiographs for three groups preoperatively and at the latest follow-up.

**Table 4:** radiographic evaluation of all included patients between groups postoperatively

Variable	Group A	Group B	Group C	*P* value
Average IC (°)	39.5 ± 4.6	39.2 ± 4.3	39.6 ± 4.6	0.882
Average FO (cm)	4.38 ± 0.52	4.23 ± 0.56	4.26 ± 0.52	0.178
The difference of bilateral FO (cm)	0.31 ± 0.21	0.32 ± 0.18	0.34 ± 0.24	0.794
LLD (cm)	0.46 ± 0.31	0.44 ± 0.34	0.44 ± 0.32	0.940

Values are expressed as mean ± SD

*P* values with statistical significance are marked with *

*IC* inclination of cup, *FO* femoral offset, *LLD* leg length discrepancy

### Complications

For the early-onset complications, 1 hip in group A and 1 hip in group C encountered delay union of the wound. And the same patients in group C also encountered hip dislocation postoperatively. With effective handling, no dislocation happened evermore and the wound recovered ultimately. Additionally, one patient in group C encountered femur fracture intraoperatively, which was fixed with several double-loop cerclage wires immediately. The patient mainly conducted function exercises in bed until the fracture healed. For this patient, he was discharged from the hospital when he mastered the function exercises in bed. He conducted follow-up as scheduled to confirm the healing status of the fracture. For late-onset complications, 12 hips (12.8%) in group A, 6 hips (12.2%) in group B, and 6 hips (11.5%) in group C encountered asymptomatic HO, all of which belonged to Brooker I or Brooker II. No significant difference was found for HO among the three groups (*P* = 0.977) and no influence was found for hip function. Other complications such as dislocation, osteolysis, and loosening were not observed at the latest follow-up.

## Discussion

In this study, cementless THA can improve hip ROM and function for AS patients with hip involvement, while no difference was found on radiological assessment for different degrees of hip involvement. The best postoperative hip ROM after THA can be realized for non-ankylosed patients. And better postoperative hip ROM can be also found on fibrous ankylosed patients than bony ankylosed ones. Moreover, Better hip function can be obtained for non-ankylosed and fibrous ankylosed patients than bony ankylosed ones.

ROM was an essential functional outcome and could be a comprehensive reflection of THA. Because for AS patients, the disease brought them not only hips involved but also ankylosed spines. The spines have little compensation in the action of squatting. So, the increase of hip ROM can improve daily activity [6]. However, the study [6] reported that hips with preoperative bony ankylosis would result in poorer postoperative ROM compared with those stiff cases. Also, our study found that different ROM improvements were realized among the three groups and the non-ankylosed hips obtained the best ROM after THA. We speculate that the soft tissue and muscles of the ankylosed hip were less-quality and weaker than that of non-ankylosed and fibrous hips due to long-term stiffness. This may result in different restoration of ROM after THA. SF-12 score including PCS and MCS was a generic health rating scale to evaluate the physical and mental health of patients [26]. And satisfaction levels reflected the clinical outcome of THA for AS patients. Lu et al. [27] have reported that the improvements in hip function and self-care ability after THA were the decisive factors in determining patient satisfaction for patients with ankylosed hips secondary to AS. But for non-ankylosed hips with some degree of mobility, pain relief and restoration of walking ability had more influence on satisfaction levels [28, 29]. And no matter non-ankylosed hips or ankylosed hips, THA can solve the problems. So, THA can realize a similar satisfaction level and SF-12 score according to our result. LOS was the first driver of cost after primary total joint arthroplasty and strategies to decrease LOS may help reduce the economic burden [30]. The increased operation time was independently associated with increased costs [31]. THA for ankylosed hip needed longer operation time and caused more surgical trauma, which may result in longer LOS and more cost. From the comparison of the non-ankylosed group with the ankylosed group, we indeed found that the less severe degree of hip involvement, the less LOS and cost were spent.

According to Pregash et al. [19], the clinical outcome of THA correlated with FO reconstruction, which was related to hip abductor strength and LLD. AS was pathologically characterized by the inflammation of tendon insertion leading to bony deformity, soft-tissue contracture, and disused weakness of abductor muscles [2, 13]. So, for AS patients, restoration of FO closed to normality and minimized LLD was crucial to good clinical outcomes. FO defined as the perpendicular distance from the center of the femoral head to the axis of the femur [32] indicated the tension of hip abductor muscles [19]. FO reconstruction can realize adequate joint stability, avoid bony and soft-tissue impingement [33, 34], and obtain the lowest liner wear as well as the least gait alteration [35]. Also, Little et al. [35] also suggested that the offset difference was also significant. They recommended [35] that if the offset difference exceeded 5 mm, the liner wear would accelerate. Although there were different surgical difficulties for the three groups, the average FO was similar. And the average FO difference was 3 mm and less than the reported 5 mm [35] for the three groups. So, for AS patients included, all hips have realized small FO differences regardless of the degrees of involvement.

LLD was also crucial for AS patients. Upadhyay et al. [36] reported that LLD was the second most common complaint from patients who were performed THA and about 8% of surgeons experienced litigation secondary to LLD. Postoperative LLD would increase frontal plane tilt angle of the pelvis and plane motion of the pelvis during walking [37] and lead to pelvis obliquity [38], gait abnormalities [39, 40], lower back pain [41], and functional disturbance of adjacent joints [42]. Besides, LLD exceeding 20 mm increased oxygen consumption [43] and LLD exceeding 10 mm increased the risk of aseptic loosening [38, 42]. For unequal leg length, the longer limb occupied 39% of the load while the shorter limb occupied 65% of the load in the static phase [44] and the bigger load for the shorter limb in the dynamic phase [45]. For AS patients, they were usually younger, more active, had higher demand for gait improvement, and cared more about the long-term survival of prostheses. So, the ambition was to realize equal limb length when performing THA for AS patients. Moreover, the detailed preoperative project and repeated confirmation intraoperatively were conducive to less LLD. As for our results, the average LLD for the three groups was not more than 0.5 cm and less than the reported 1 cm [38, 42]. And no difference was found among the three groups.

The study reported that AS patients showed a higher rate of perioperative and postoperative complications after THA including wound complication, polyethylene wear, revision, and dislocation [46]. Survival analysis of THA for AS was 81.4% at 15 years [4], which was lower than that of THA for other etiologies [47]. Also, the rate of polyethylene wear of THA for the bony ankylosed hip was high than that of primary THA concluded by Kim et al. [48]. Because the AS patients receiving THA were usually younger and more active. They had a high demand for hip function and the activity level was also higher than patients with osteoarthritis [46]. Additionally, AS was characterized by the spine and hip joint involved. The patients were usually accompanied with abnormal spinopelvic mechanics leading to abnormal stress to the implants and abnormal positions of the pelvis for AS patients increasing the difficulties of inserting the prosthesis in ideal positions [10, 49]. So, more activity and possibly suboptimal component position can account for the higher postoperative complications. HO was another complication encountered after THA for AS patients but was symptomatic in a minority of patients [50]. According to the published literature, 10.5% of patients developed HO consistent with Brooker I or II and no reports of re-ankylosis [51]. And nearly 12% of patients with ankylosed hips may encounter HO belonging to Brooker I [52]. The incidence of HO in our study was nearly 12% and classified as Brooker I or II. And no influence was found on hip function. This was similar to the published result.

We noted some limitations of the study. This was a retrospective study in a single joint center with the unavoidable weakness inherently. But the study was the currently biggest sample-size research on the outcomes of THA for hip involved secondary to AS. Also, we firstly compared the effectiveness of THA for three different degrees of hip involvement. The surgery-specific information including perioperative blood loss, surgery time, and inflammatory response to surgical trauma can’t be obtained. However, with the fast development of enhanced recovery after surgery, the blood loss, traumatic response, and surgical time can be lesser and lesser. At last, we can’t give accurate information about the AS duration and symptom duration. Because most of the included patients were not treated in our hospital before surgery. And we can’t obtain accurate preoperative information.

## Conclusion

For AS patients with three different degrees of hip involvement, THA can improve hip ROM and enhance hip function. The better hip ROM and clinical outcomes postoperatively can be realized for the non-ankylosed hips than ankylosed ones with relatively less cost and LOS.

## Data Availability

The datasets used and/or analyzed during the current study are available from the corresponding author on reasonable request.

## Abbreviations

AS: Ankylosing spondylitis
ROM: Range of motion
THA: Total hip arthroplasty
HHS: Harris hip score
SF-12: The short-form 12 health survey
LOS: Length of stay
THE: Total hospital expense
LLD: Leg length discrepancy
HO: Heterotopic ossification
PCS: Physical component summary
MCS: Mental component summary
IC: Inclination of cup
FO: Femoral offset
ANOVA: A 1-way analysis of variance
HLA B27: Human leukocyte antigen B27
CoC: Ceramic-on-ceramic
CoP: Ceramic-on-polyethylene
